# Dogs' looking times and pupil dilation response reveal expectations about contact causality

**DOI:** 10.1098/rsbl.2021.0465

**Published:** 2021-12-22

**Authors:** Christoph J. Völter, Ludwig Huber

**Affiliations:** Comparative Cognition, Messerli Research Institute, University of Veterinary Medicine Vienna, Medical University of Vienna and University of Vienna, Veterinaerplatz 1, 1210, Vienna, Austria

**Keywords:** comparative cognition, causal perception, animacy cues, physical cognition, eye tracking‌, canine cognition

## Abstract

Contact causality is one of the fundamental principles allowing us to make sense of our physical environment. From an early age, humans perceive spatio-temporally contiguous launching events as causal. Surprisingly little is known about causal perception in non-human animals, particularly outside the primate order. Violation-of-expectation paradigms in combination with eye-tracking and pupillometry have been used to study physical expectations in human infants. In the current study, we establish this approach for dogs (*Canis familiaris*). We presented dogs with realistic three-dimensional animations of launching events with contact (regular launching event) or without contact between the involved objects. In both conditions, the objects moved with the same timing and kinematic properties. The dogs tracked the object movements closely throughout the study but their pupils were larger in the no-contact condition and they looked longer at the object initiating the launch after the no-contact event compared to the contact event. We conclude that dogs have implicit expectations about contact causality.

## 1. Introduction

Identifying causal relations in the environment is notoriously challenging. However, watching certain spatio-temporally contiguous object interactions such as launching events consistently leads to a causal impression in humans [1]. Habituation–dishabituation studies have provided evidence for such causal perception already in the first year of life [2]. In regular launching events, one object (A) approaches another object (B) and when the two objects make contact, object B immediately starts moving, whereas object A stops. Infants from the age of six to seven months react with longer looking times in control trials without collision events (i.e. the objects move in the same way as in a launching event but a gap remains between them) or with a delay (i.e. when object A collides with object B there is a delay before object B starts moving) after having been habituated to normal launching events [3–5]. They also looked longer at normal launching events (but not at novel delayed or no-collision control events) after having been habituated with no-collision or delayed events [4,5]. Sensitivity to spatio-temporal contiguity is, therefore, early developing in humans though its postulated innateness or modularity remain controversial [2,6,7].

While causal perception has received a lot of attention in the human developmental literature, we know surprisingly little about it in non-human animals. It has been claimed that tool-using species especially might form expectations about contact causality [8]. Indeed, there is evidence that non-human great apes are sensitive to contact principles in tool selection tasks [9,10]. Chimpanzees (*Pan troglodytes*) also looked longer at videos of object interactions that violated the contact principle (e.g. a hand appearing to lift a banana from a distance without making contact with it) compared to control events (the hand grasping and lifting the banana) [11]. Only one study investigated collision events in a comparable way to human studies by using sparse and simple visual stimuli; the authors found some evidence that chimpanzees process colliding two-dimensional stimuli in a similar way as humans [12]. Outside the primate order, newly hatched chicks (*Gallus gallus*) have been found to show a preference for approaching self-propelled objects over objects that were passively moved in a launching event but control conditions including delayed or no-collision events are missing.

Dogs are a promising species to examine whether non-tool using species are sensitive to contact causality (e.g. [13–16]). Recent evidence from violation-of-expectations (VoE) paradigms suggest that they form implicit expectations about certain physical properties including solidity [17], size constancy [18,19] and object permanence [20]. The VoE paradigm compares participants' reactions to novel events that are either consistent or inconsistent with some environmental regularity. If participants have expectations about this regularity, they should show a surprise response towards the inconsistent event. In the context of VoE studies with infants, pupil dilation has been suggested as a superior response variable compared to looking time measures owing to the temporal sensitivity of the phasic pupil dilation response and its stability to test-order effects [21,22]. Few eye-tracking studies have so far examined pupil size data in dogs. These studies showed that dogs exhibited dilated pupils when presented with pictures of (male) human faces with an angry emotional expression compared to a happy expression [23,24].

In the current eye-tracking study, we presented dogs with realistic three-dimensional animations of moving balls. After a familiarization, the dogs saw two new test events: launching events with contact or without contact between the involved objects. We hypothesized that the dogs would find the no-contact condition more surprising than the contact condition. Accordingly, we predicted larger pupils and longer looking times in response to the no-contact than the contact condition.

## 2. Methods

### (a) Subjects

We tested 14 pet dogs (five border collies, five mixed breeds, two Labrador Retrievers, one collie, one Australian Shepherd; mean age: 30.6 months, range: 13–79 months; eight females, six males).

### (b) Stimuli

We presented the dogs with a familiarization video and two different test videos. The videos (frame rate: 100 fps) had a duration of 3.7 s but we extended presentation time of the last video frame in the experiment for a total video duration of 4.5 s in familiarization trials and 13.5 s in test trials.

**Figure 1.**
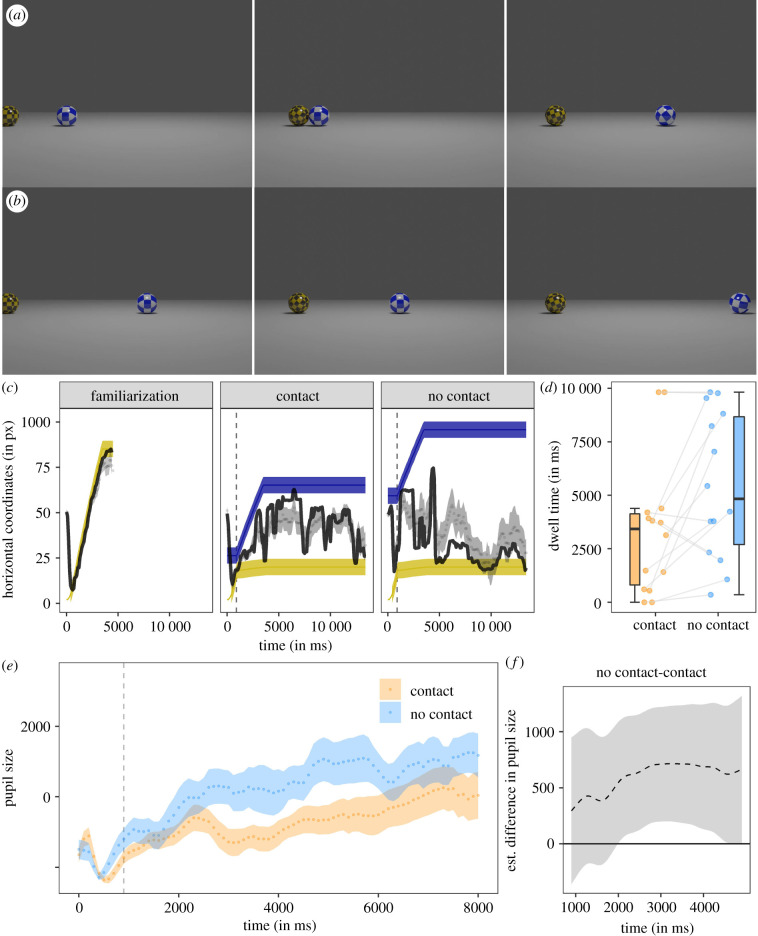
Screenshots of the contact condition (*a*) and the no-contact condition (*b*) at 0, 920 and 3700 ms. (*c*) Time-series plot showing the dogs' median (black line) and mean horizontal gaze coordinates (± s.e. dotted line and dark grey shaded area) in the final familiarization trials and in the test trials. The shaded yellow and blue areas show the position of the launching and target ball. The dashed vertical line indicates the time when the target ball started moving (also in *e*). (*d*) Box plot showing the dogs’ looking times in the interest areas around the launching ball at the end of the video. The dots represent the individual looking times. (*e*) Time-series plot showing dogs' pupil size (in arbitrary units and baseline corrected). The orange and blue lines show the mean pupil size (± s.e.) in the contact and no-contact condition. (*f*) Difference curve derived from GAMM01. The dashed line shows the estimated difference between the no-contact and contact condition; the shaded area shows the pointwise 95% CI.

The familiarization video showed a yellow-black patterned ball rolling along a grey surface from left to right. It got slower towards the right edge of the screen and stopped before it moved outside of view. In the test videos, there were two balls: again the yellow-black patterned ball starting on the left side of the screen (henceforth: launching ball) and a blue-white patterned ball (henceforth: target ball) closer to the centre of the screen. The videos started with the launching ball rolling towards the inactive target ball. In the contact condition (figure 1*a*), the target ball was located closer to the launching ball than in the no-contact condition (figure 1*b*). In the contact condition, the launching ball hit the target ball, thereby, setting the latter into motion (while the launching ball abruptly stopped moving). The target ball rolled towards the right edge of the screen and stopped moving before it moved out of view. In the no-contact condition, the launching and target balls moved with exactly the same kinematics as in the contact condition. However, because the two balls were further apart, the launching ball abruptly stopped moving and the target ball was set into motion without any contact between the two balls (or any other obvious cause; see the electronic supplementary material, movie S1).

### (c) Design and procedure

Each subject was presented with two test conditions, the contact and no-contact conditions. The order of conditions was counterbalanced across subjects. We pseudo-randomly assigned the dogs to the order groups and balanced the groups as much as possible with respect to age, sex and breed. We conducted two sessions per dog (minimum intersession interval: 6 days to reduce the likelihood for carry-over effects between conditions). Each session consisted of three identical familiarization trials followed by one test trial.

We used the EyeLink1000 eye-tracking system to record the dogs' eye movements and pupil size (see the electronic supplementary material for details).

### (d) Analysis

To analyse to what extent the dogs followed the movements on the screen, we calculated, separately for each ball (launching and target) and condition, the proportion of variance in dogs' horizontal gaze positions (*r*^2^-values) explained by the horizontal ball coordinates for the period starting 500 ms after the onset of the video until the end of the movement of the ball (for the target ball we used the onset of the target ball motion as a starting point). We fitted a linear mixed model with the dogs' horizontal gaze positions at every millisecond (ms) as the response variable, the *x*-coordinates of the ball centre as the predictor variable and subject identity as random intercept. The *r*^2^-values provided a numerical measure of how well the dogs followed the movement (there is no statistical interpretation beyond that).

To examine differences in looking times across conditions, we analysed the interest period at the end of the video when the last frame of the video was shown for 9.8 s. We defined interest areas (IA) around the end positions of the launching and target ball (w × h: 240 × 400 px). We compared the looking time in each IA across conditions using two-tailed, paired-samples *t*-tests.

The pupil size data were pre-processed [25] by excluding samples within 100 ms of blink events, applying a linear interpolation and conducting a subtractive baseline correction. We used the entire 900 ms pre-event period (i.e. before the target ball started moving) as baseline. Finally, we sampled the data down to 10 Hz to reduce autocorrelation.

For the pupil size analysis, we fitted a generalized additive mixed model (GAMM; implemented using the R function 'bam' of package 'mgcv' [26]; see the electronic supplementary material) with Gaussian error structure to the pre-processed pupil size data [27,28]. We analysed a 4 s interest period starting at the end of the baseline period. We used smoothing parameter selection method ‘ML'. In GAMM01, we included condition (contact or no-contact) as a factor, the non-parametric regression lines for time (every 100 ms after the collision event) and the interaction between condition and time (with the upper limit for the number of knots set to 20), and the non-parametric interaction between *X* and *Y* gaze positions (given that the gaze position can affect the pupil size [25,28]). We also included non-parametric random effects (random factor smooths) for each individual time-series trajectory (i.e. for each subject and test trial) to improve the model fit and to account for autocorrelation [27,28]. All data files and R scripts are available on Zenodo [29] and Dryad [30].

## 3. Results

The dogs looked consistently on the screen while the video was playing (proportion of on-screen looking time: familiarization: median: 0.92, range: 0.75–0.97; contact: median: 0.93, range: 0.88–0.97; no-contact: median: 0.93, range: 0.85–0.99). Their on-screen looking time did not differ significantly between the two test conditions (*t*_13_ = −0.79, *p* = 0.442). In the familiarization, dogs followed the rolling ball closely (final familiarization trials: *r*^2^: 0.80; figure 1*c*). In the test trials, the dogs for the most part also followed the movement of the launching ball while it was moving in both conditions (contact: *r*^2^: 0.86; no-contact: median *r*^2^: 0.73). When the target ball started moving some dogs also looked back and forth between the balls, which led to smaller *r*^2^-values for the target ball (contact: *r*^2^: 0.44; no-contact: *r*^2^: 0.57).

### (a) Looking time analysis

Despite large individual variation in looking times (figure 1*d*), we found that the dogs looked significantly longer at the launching ball end position IA (same position on the screen across conditions) in the end interest period (when the balls did not move anymore) in the no-contact condition compared to the contact condition (*t*_13_ = −2.66, *p* = 0.020, 95% confidence interval (CI) (392, 3797)). When comparing the looking times to the respective end positions of the target ball, we found longer looking times in the contact than the no-contact condition (*t*_13_ = 2.30, *p* = 0.039, 95% CI (−4048, −123)).

### (b) Pupil size analysis

The comparison between GAMM01 and a null model without the condition factor and the non-parametric regression lines of the condition levels over time indicated that condition significantly improved the model fit (chi-square test on the difference in marginal likelihood scores: $\chi_{5}^{2}=7.54$, *p* = 0.010; GAMM01 had a lower Akaike information criterion (AIC): *Δ*AIC 19.98). The model summary revealed that dogs' pupils were significantly larger in the no-contact condition compared to the contact condition (*t* = 2.43, *p* = 0.015; see the electronic supplementary material, table S1). The difference curve showed that the pupil size differed significantly between conditions in the time window between 1991 and 4900 ms starting approximately 1000 ms after the onset of the target ball motion (figure 1*e*; for the model estimates and partial effects see the electronic supplementary material, table S2 and figure S3). We also found evidence for a significant change of the pupil size over time in the no-contact condition (*F*_9.00,11.07_ = 2.10, *p* = 0.013) but not the contact condition (*F*_1.18,1.23_ = 2.04, *p* = 0.141). An analysis of aggregated pupil size data confirmed the significant difference between conditions and provided no evidence that the order of conditions had an effect on performance (see the electronic supplementary material).

## 4. Discussion

In line with our prediction, dogs had larger pupils and looked longer at the launching ball in response to the no-contact event than the contact event. This finding provides, to our knowledge, the first evidence that dogs are sensitive to the principle of contact causality underlying such launching events.

Looking times and the psychosensory pupil response are only indirectly linked to cognitive abilities or mental states, and they are unspecific indicators, i.e. many factors can lead to similar looking times or pupil size responses [31]. Thus, other factors such as low-level perceptual aspects might provide alternative explanations. However, the animations used in this experiment were identical with respect to depicted visual elements and their kinematic properties and we controlled for the dogs’ gaze position in our analysis. Moreover, changes in the pupil size might stem from differences in dogs' general attentiveness between conditions. However, we found no evidence for differences in dogs’ overall on-screen viewing times and they followed the movements of the balls closely in both conditions.

The difference in dogs' reaction to the conditions might relate to the unexplained sudden stop of the launching ball in the no-contact condition, the sudden unexplained start of the target ball movement, or both. The looking time analysis at the end of the video indicates that the dogs looked longer at the launching ball in the no-contact than the contact condition and *vice versa* for the target ball end position. The longer looking times to the target ball in the contact condition might simply reflect a preference for the stimulus that they saw moving last. However, in the no-contact condition the dogs deviated from the assumed preference for the last moving object. In line with the causal perception hypothesis, the impression of the launching ball setting the target ball into motion from a distance might have increased the dogs’ interest in the launching ball.

Dogs’ sensitivity to contact causality might hint towards a wide phylogenetic distribution of causal perception among mammals, not restricted to tool-using species. Additionally, the pet dogs' experience with human artefacts (including balls) might have contributed to their expectations about how these objects behave when they collide. These two possibilities are not mutually exclusive and future research with different dog populations (that might have less experience with such artefacts) and non-domesticated species (e.g. wolves) will help to clarify the contribution of nature and nurture on causal perception in canids and mammals more generally.

In summary, our findings are consistent with the notion that dogs form implicit expectations about object interactions involving contact causality. At a methodological level, our study highlights the potential of pupillometry for the investigation of expectancy violations in dogs. Future studies should also examine whether temporal lags between the contact event and the onset of the target motion can induce a pupil dilation response in dogs.

## Data accessibility

The data and R scripts associated with this manuscript are available on Zenodo (https://doi.org/10.5281/zenodo.5723047) and Dryad (https://doi.org/10.5061/dryad.9ghx3ffj8) [30]. We provided a description of our dataset together with the raw data files on Zenodo and Dryad.
